# Social influence and academic performance: a serial mediation model in AI-enhanced learning

**DOI:** 10.3389/fpsyg.2026.1928799

**Published:** 2026-09-18

**Authors:** Anish Kumar Bhunia, Saeed Alzahrani

**Affiliations:** 1 Department of Management & Humanities, National Institute of Technology, Arunachal Pradesh, Jote, Arunachal Pradesh, India; 2 Department of Management Information Systems, College of Business Administration, King Saud University, Riyadh, Saudi Arabia

**Keywords:** academic performance, academic stress, AI self-efficacy, higher education, mental wellbeing, Saudi Arabia, serial mediation, smart learning

## Abstract

**Introduction:**

This study examines how social influence affects the academic performance of Saudi university students, focusing on the mediating roles of AI self-efficacy, smart learning, academic stress, and mental wellbeing. The study is grounded in Social Learning Theory.

**Methods:**

A quantitative research design was employed using Partial Least Squares Structural Equation Modeling (PLS-SEM). Data were collected from 345 university students enrolled at three public universities in Saudi Arabia. A theoretical model based on Social Learning Theory was developed to examine the relationships among social influence, AI self-efficacy, smart learning, academic stress, mental wellbeing, and academic performance.

**Results:**

The findings supported all 10 proposed hypotheses. Social influence had a significant direct effect on academic performance (*β* = 0.156). Significant indirect effects were also observed through AI self-efficacy(*β* = 0.223), smart learning (*β* = 0.050), academic stress (*β* = 0.032), and mental wellbeing (*β* = 0.064). The serial mediation pathway was also significant (*β* = 0.003). All mediation effects were partial, indicating that social influence influences academic performance through both direct and indirect pathways. Among the constructs examined, AI self-efficacy was the strongest predictor of academic performance (*β* = 0.331).

**Discussion:**

The findings show that social influence affects academic performance through multiple psychological and learning-related pathways. AI self-efficacy emerged as the strongest predictor, highlighting the importance of students’ confidence in using AI for learning. Overall, the study extends Social Learning Theory to AI-supported higher education and emphasizes the importance of AI self-efficacy, smart learning, and psychological wellbeing in promoting academic success among Saudi university students.

## 1 Introduction

Student dropout and academic failure have become major challenges for universities across the world, including in the United States and Europe (Berlingieri and Bolz, 2025), Spain (González-Morales et al., 2025), and Indonesia (Cai and Meng, 2025). The United States alone witnesses a shocking 40% college dropout rate per annum. According to the most recent statistics, 39% of students do not achieve their bachelor’s degree within 8 years, and 22.3% drop out during their first year. On average, college dropouts earn 35% less than those who have a degree, and they are twice as likely to be unemployed (Cai and Meng, 2025). This issue is also present in the Kingdom of Saudi Arabia, where universities face similar problems with student disengagement. Statistics illustrate that dropout rates in Saudi universities are highly alarming. On an aggregate basis, between 20 and 68% of students drop out of Saudi higher education, with this wide range varying by field. It is a problem with wider social consequences, including high unemployment and increased social stigma (Abdulghani et al., 2023; Alhamad and Singh, 2024; Siddiqui et al., 2025).

Recent studies have identified mental health and AP as key factors contributing to university dropout rates. University students often face various personal, social, and academic challenges that can substantially affect their mental wellbeing and AP (AP, hereafter) (Chongjin et al., 2025). Understanding the determinants of students’ performance in university-level education is of significant concern to educational institutions. This concern has led to a high volume of research on the subject, resulting in the establishment of journals on the topic. The main purpose of such research is to understand the factors affecting AP to devise strategies for improving performance.

AP refers to how well students, teachers, or educational institutions achieve short- and long-term learning goals, and it is usually assessed through tests, assignments, or grades (Hailu et al., 2024). It is now recognized as a complex construct that involves not only knowledge acquisition but also the development of skills and competencies, shaped by cognitive, motivational, and emotional factors across different educational contexts. It is critical to understand AP, as it significantly determines individual outcomes such as job prospects, socioeconomic mobility, and broader societal and economic development (Ruiz-Ortega and Berrios-Martos, 2025).

The literature has so far reviewed the factors known to influence AP, such as demographic, technological, social, psychological, institutional, and financing policy–related determinants (Cai and Meng, 2025; Duc, 2026; Hayat et al., 2020; Lourenço et al., 2025). Over the last few years, social influence (SI) and the increasing use of Artificial Intelligence (AI) have had a significant impact on the lives of ordinary people and on how people carry out conversations, interact with others, and find information. This transformation is more pronounced among youths who find themselves in educational institutions where AI has been embedded in their curriculum and in their regular social routine. Nevertheless, the prevalence of these influencers on the correlation between learning, academic stress, mental wellbeing, and AP continues to be ignored despite much research into other areas; hence, there is a need for further research work (Bays et al., 2023; Shahzad et al., 2024; Chongjin et al., 2025).

SI is widely recognized as a key factor in students’ AP. SI is the degree to which individuals perceive those important others (e.g., family, friends, peers, or instructors) influence their attitudes, decisions, and behaviors. Supportive SI are most often associated with high academic achievement, while negative peer influences can affect students’ academic progress (Shao et al., 2024).

AI Self-efficacy (ASE) has recently been recognized as a key psychological variable that affects the AP of university students. ASE is an individual’s confidence in their ability to effectively use artificial intelligence (AI) technologies to accomplish learning tasks and solve problems. Today, AI is not used in education just to deliver learning outcomes through personalized instruction, simplified administrative processes, and directed academic support. As AI becomes more prevalent in society and in the workforce, students should possess ASE and confidence in their ability to operate/understand AI tools competently. This kind of confidence increases motivation, persistence, and achievement, helping students remain involved and prepared for success in an AI-driven world (Bewersdorff et al., 2025).

Additionally, another psychological determinant of university students’ AP is Academic Stress (AS). It is the mental pressure students experience academically when there are many assignments, and they lack the time or skills to complete them. It is one of the main sources of stress in students’ lives. Writing tests, having many exams in the same week, and trying to perform well in courses are some of the high-anxiety activities for college students. Research shows that stress influences how students think, understand information, remember things, and perform academically. Superior academic stress decreases active learning, depresses AP, causes emotional tiredness, and even drives learners from their studies (Akgun and Ciarrochi, 2003; Pritchard and Wilson, 2003; You, 2018).

Furthermore, Smart Learning (SL), as one of the technological–pedagogical constructs, affects AP in a specific way: it enables, nurtures, and empowers learners to access and use AI and social networking in their learning activities (Allal-Chérif et al., 2021; Muro et al., 2018). SL is the use of intelligent digital technologies and adaptive learning environments to enhance learning efficiency, engagement, and academic outcomes. They not only afford real-time learning but also encourage learner-to-content, learner-to-learning, and learner-to-teacher interactions. This affords easy access to learning resources, supports collaboration, and increases students’ self-reliance and effectiveness (Shahzad et al., 2024; Samaha and Hawi, 2016).

Moreover, Mental Well-Being (MWB) is another psychological construct that influences AP. It refers to a balanced state of effective psychological functioning and positive emotional health, and has a great impact on wellbeing and AP (Annamalai et al., 2020). Positive MWB makes students more focused, motivated, and resilient, which, in turn, improves their AP. On the other hand, negative mental states and stress impair concentration and engagement, which could lead to poor results and dropout. Supporting students’ MWB helps them effectively manage stress, think more creatively, and achieve academic success (Shengyao et al., 2024).

These factors have been exhaustively addressed in Western societies and in some parts of Asia. However, very little has been done on the determinants of AP among Saudi students, suggesting that much more investigation is needed in this area. This gap indicates that research is needed to determine whether there are structural relationships among SI, ASE, SL, AS, MWB, and AP, as, to the best of our knowledge, none of the studies have yet considered all these variables within a single model to explore their combined impact on students’ AP.

The paper makes two key contributions—theoretical and practical. Theoretical contribution is the development of an integrated model (see Figure 1), based on the principles of Social Learning Theory (Bandura and Walters, 1977) in order to explain relationships between the proposed variables and academic performance of students, which fills the existing research gap and serves as the basis for further research. Practical contribution lies in the provision of recommendations to educators, administrators and policy makers on how to facilitate AI-enabled learning, develop students’ self-efficacy regarding AI usage and improve their psychological wellbeing and academic performance.

**Figure 1:**
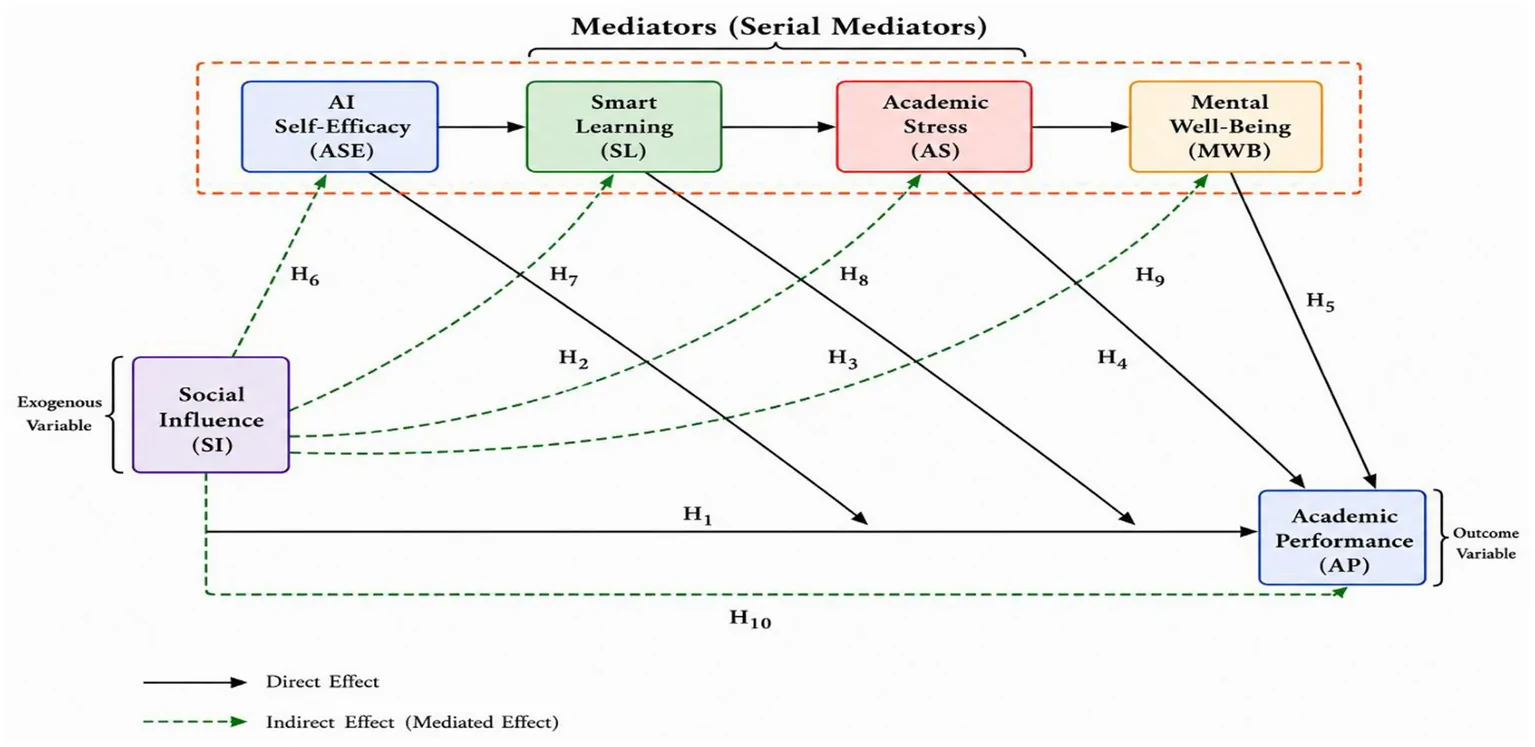
The proposed research model.

## 2 Conceptual foundations and literature review

### 2.1 Social learning theory (SLT)

The linkage between the six variables and Social Learning Theory (SLT) can be described as follows: According to the SLT, people adopt behaviors, attitudes, and skills through social interactions, observation, and imitation (Bandura and Walters, 1977). Considering this social context, SI plays a leading role in shaping students’ perceptions and reinforcing their motivation to adopt behaviors observed from peers, lecturers, and others. That’s why AI tools for learning will be among such behaviors to be observed. After repeated observations and successes, learners acquire AI self-efficacy, a measure of their belief in their ability to effectively use AI technologies (Bewersdorff et al., 2025; Shahzad et al., 2025). These competences learned socially form the foundation for smart learning, as students copy technology-enabled learning methods demonstrated by their peers and academic environment (Shahzad et al., 2024). Also, SLT shows how smart academic settings can lessen academic stress and promote psychological wellbeing, as new academic pressures on students may make it hard for them to get used to a new environment, which in turn may lower motivation, affect AP, and raise the dropout rate. Taken together, socially learned behaviors enhance students’ AP as learners apply effective strategies observed in others and reinforced by societal feedback and institutional norms (Almoslamani, 2022).

### 2.2 SI and AP

In this study, SI is defined as graduate students’ belief that people around them, such as faculty members and peers, encourage and support them in using AI (Uludağ et al., 2025). SI plays a vital role in students’ AP. Students’ AP is largely affected by peer groups’ performance and social influence. A positive and supportive social environment helps students to adopt better learning strategies and improves AP (Wolor and Swaramarinda, 2024). Similarly, a supportive peer network and connections with successful students are associated with better academic outcomes (Salari et al., 2025; Yu et al., 2026). Healthy teacher-student relationships and peer relationships are also associated with better academic success (Mieziene et al., 2022; Murray-Harvey, 2010; Lavy and Sand, 2012; Oh et al., 2020). Furthermore, emotional and social support from parents, teachers, and peers helps alleviate stress and improve mental health and learning outcomes. Likewise, in a digital learning environment, SI and supportive teacher interactions are vital because they help create a sense of social presence and facilitate learning (Singh et al., 2022; Qi and Derakhshan, 2025; Alshammari and Alkhwaldi, 2025). Although a study did not find a direct correlation between SI and AP (Shin, 2003) recent studies have shown that SI has a significant positive impact on AP (Zhang and Qian, 2024). Based on this, the following hypothesis is proposed:

*H1*: SI has a positive predictive relationship with AP.

### 2.3 ASE and AP

AI self-efficacy is the general belief of individuals in their ability to use and interact with AI. In the educational domain, we define AI self-efficacy as students’ confidence not only in their ability to use and interact with AI technologies but also in their ability to comprehend and learn about AI technologies and applications (Wang and Chuang, 2024). AI has become a highly influential factor in the field of education, greatly impacting individual learning outcomes. Previous studies have established that AI positively impacts academic results by providing individualized learning opportunities, facilitating administrative tasks, and providing support mechanisms (Yu, 2023). ChatGPT, a generative AI tool, provides immersive learning opportunities for students. The tool provides a real-world learning environment, allowing students to apply their knowledge practically and improve their cognitive skills. By providing interactive learning opportunities, AI, as represented by ChatGPT, Google Gemini, DeepSeek, Anthropic Claude, Microsoft Copilot, and Perplexity AI, has the potential to impact academic results (Shahzad et al., 2024; Pang, 2024). For example, personalized learning based on students’ abilities and interests can improve engagement and understanding. However, the rapid development of AI may undermine users’ self-efficacy because of the constant need to improve one’s competence. Such constant pressure may cause users to feel stressed and develop negative beliefs about AI, thus undermining their self-efficacy. In addition, the stress of using technology may make users less willing to use it for educational purposes. Recent studies show mixed results about AI’s impact on AP. Some find little or no effect (Be’cirovi’c et al., 2025), while others report positive (Shahzad et al., 2024; Makhambetova et al., 2021; Zhai et al., 2024) or even negative (Cambra-Fierro et al., 2025; Rodway and Schepman, 2023) effects. Accordingly, the following hypothesis is formulated:

*H2*: ASE has a positive statistical effect on AP.

### 2.4 SL and AP

Smart learning is a technology-enabled, personalized learning approach that uses digital tools and data to enhance learning effectiveness, engagement, and flexibility (Allal-Chérif et al., 2021). SL helps to develop a personalized and effective self-learning experience by using AI and technology to improve students’ access to learning resources, thus improving AP. SL also helps educational institutions to develop a positive and effective learning environment by improving students’ engagement, collaboration, and AP. In the context of a university, SL tools help to develop a positive and effective learning environment by fostering a supportive and conducive atmosphere for the development of students and their mental wellbeing (Vedøy et al., 2020). AI-based SL tools help to develop a positive and effective learning environment by fostering a supportive learning community that enhances students’ self-efficacy and feelings of belonging. Generative AI technologies help to develop a positive and effective learning environment by fostering collaborative knowledge construction and dialogue, thus enhancing SL. ChatGPT, Google Gemini, DeepSeek, Anthropic Claude, Microsoft Copilot, and Perplexity AI tools help to develop a positive and effective learning environment by fostering active learning and instant interaction and academic support, thus enhancing AP (Shahzad et al., 2024; Beerbaum, 2023). Although the goal of the use of SL is to improve AP, empirical studies suggest that there may be detrimental effects on AP as well, such as reduced self-efficacy, increased cognitive overload, and reduced grades (Mantooth et al., 2021). Based on the above discussion, the following hypothesis is proposed:

*H3*: SL has a positive association with AP.

### 2.5 AS and AP

AS is a psychological strain that results from the high academic demands and the corresponding lack of resources, including time and personal capacity. Studies show that AS significantly affects cognitive appraisal, information processing, memory, and performance. University students may experience academic pressures, such as the number of examinations conducted over short periods and the need to perform well in their academic courses, which may affect their resilience and consequently AP (Becker and Börnert-Ringleb, 2025; Bin Abdulrahman et al., 2025). A high academic workload and academic curriculum may also increase AS, which may result in compromised AP, health, and behavioural changes (Pascoe et al., 2020; Al-Roomy, 2025). irrespective of factors including gender, role, race, and academic standing (Barbayannis et al., 2022). However, the findings concerning the relationship between AS and AP have not been entirely consistent. The majority of the studies found a negative impact (Pascoe et al., 2020; Al-Roomy, 2025; Iqra, 2024; Lai and Ng, 2026; Deng et al., 2022), some found no impact (Cheng and Catling, 2015; Petrie and Stoever, 1997; Zhang Y. et al., 2024), and a few found a slight positive impact (You, 2018; Zajacova et al., 2005). In light of the above discussion, the following hypothesis is developed:

*H4*: AS has a negative association with AP.

### 2.6 MBW and AP

MBW is a state of mental health in which a person can think clearly and experience balanced emotions (Annamalai et al., 2020). MWB has long been a concern among university students worldwide. It is influenced by factors such as changes in instructional modes, learning resources, social supports, and the educational environment. This is particularly relevant in the context of management education, as the curriculum is highly demanding and students must develop a range of personal and professional competencies within a limited time frame (Chaudhry et al., 2024; Mishra and Nargundkar, 2015). MBW helps the student adjust to life at university and encompasses self-acceptance, positive relationships, autonomy, mastery over the environment, personal growth, and purpose in life (Díaz et al., 2006). This period in the student’s life is stressful due to new learning environments and increased academic pressure, making it a critical period with higher anxiety and lower wellbeing compared to the general population (Cooke et al., 2006; Pérez et al., 2019). MBW in the first semester is critical to preventing sub-optimal AP throughout the undergraduate program (Chu et al., 2022). MBW is a strong predictor of AP (Podsakoff and Organ, 1986). Elevated wellbeing is associated with better academic outcomes through student engagement, motivation, and concentration, whereas reduced wellbeing is associated with dropout and poor AP (Agha et al., 2023; Reuter and Forster, 2021; Kilgore et al., 2023). MBW may also be linked to other constructs such as empathy, kindness, and patience in young adults (Korda et al., 2025). However, the literature is mixed regarding the link between MWB and AP. Some studies found a positive correlation (Li et al., 2022; Chu et al., 2022; Monzonís-Carda et al., 2025), while some found both positive and negative correlations (Rüppel et al., 2015). Based on the preceding discussion, the following hypothesis is formulated:

*H5*: MBW is positively linked with AP.

### 2.7 Mediating role of ASE

SI is found to be a significant factor in the adoption and utilization of AI technologies by scholars, with a positive impact on their intentions to engage in AI processes and on the propagation of SI in the social network (Acosta-Enriquez et al., 2025). SI, through observational learning and social comparison, increases students’ positive expectations about the use of AI applications, facilitated by instructors and peers (Van den Broeck et al., 2023). ASE refers to confidence in the effective application of AI technologies, with tools such as ChatGPT and DeepSeek facilitating learning and self-confidence development through interactive platforms, personalization, and instant feedback. Given the strong link between ASE and AP, a greater SI is likely to boost ASE, thereby enhancing AP (Shahzad et al., 2025;Singh et al., 2025). Based on the above discussion, the following hypothesis is proposed:

*H6*: ASE statistically mediates the association between SI and students’ AP.

### 2.8 Mediating role of SL

The social constructivist theories assert that SI play an integral part in the learning process. The theories emphasize the importance of SI in shaping the learning process. The theories assert that cognitive development and understanding can be enhanced through collaborative discourses and discussions (Gherghel et al., 2023; Gao et al., 2024). SI, particularly the interaction among learners and between learners and instructors, has become an essential element of the learning process. Recent studies have shown that learners can receive feedback from one another through bilateral communication, such as sharing ideas and discussing, which improves their learning outcomes (Yanqing Wang et al., 2022; Pan et al., 2024). Educational institutions are making extensive use of SL to increase student engagement, foster collaboration, promote mental wellbeing, and boost AP (Boer et al., 2021). A foundational study found that SL mediates the relationship between SI and students’ AP (Shahzad et al., 2024) In light of the above discussion, the following hypothesis is suggested:

*H7*: SL statistically mediates the association between SI and students’ AP.

### 2.9 Mediating role of AS

SI in the form of social support has been linked to lower levels of AS. This highlights the importance of the social setting and family support in fostering a conducive, positive psychological environment for learning. SI is the attention and assistance received from family, friends, or staff at the institution to help cope with AS (Puteri and Dewi, 2021). The research consistently demonstrates that SI serves as a significant protective factor against AS. This occurs through both direct psychological benefits and by buffering the negative impacts of academic stressors. Academic pressure experienced by university students may stem from the clustering of examinations over short periods and the need to attain high standards in academic subjects, which may affect resilience, thereby affecting AP (Becker and Börnert-Ringleb, 2025; Bin Abdulrahman et al., 2025). A heavy academic load and curriculum may also increase AS, negatively affecting AP, health, and behavior. Most studies have revealed that AS negatively impacts AP (Pascoe et al., 2020; Al-Roomy, 2025; Iqra, 2024; Lai and Ng, 2026; Deng et al., 2022). Based on the reviewed literature, SI may act as a protective factor that prevents AS, thereby enhancing AP.

*H8*: AS statistically mediates the association between SI and students’ AP.

### 2.10 Mediating role of MBW

SI, as a form of conformity, compliance, and obedience, significantly affects MWB by shaping individuals’ behaviors, beliefs, and emotions within a social group. Positive and supportive SI meet individuals’ psychological needs for competence and relatedness, whereas negative social influence may lead to anxiety, identity suppression, and cognitive dissonance (Umberson and Montez, 2010). Moreover, MBW is a strong predictor of AP. Higher wellbeing is related to better academic performance by way of increases in student engagement, motivation, and concentration, whereas decreased wellbeing is related to dropout risk and poor AP (Agha et al., 2023; Reuter and Forster, 2021; Kilgore et al., 2023). It may be argued that high levels of social influence promote psychological wellbeing, which in turn serves as a catalyst for high levels of academic achievement (Gai et al., 2026).

*H9*: MBW statistically mediates the association between SI and students’ AP.

### 2.11 The serial mediating role of ASE, SL, AS, and MBW

Based on the reviewed literature, it can be established that AP can be enhanced by SI through four mediating factors. To begin with, SI can promote SL through collaborative discussions with others (Shahzad et al., 2024). The promotion of SL can then improve MBW (Hu et al., 2026). The improvement in MBW can then allow the student to effectively utilize AI tools, thereby improving ASE (Marques-Pinto et al., 2025). The improvement in ASE can then enable the student to meet the demands of their work more efficiently, thereby reducing AS that may result from excessive workloads. The reduction in AS can then improve AP, as AS has a negative impact on AP (Pascoe et al., 2020; Al-Roomy, 2025; Iqra, 2024; Lai and Ng, 2026; Deng et al., 2022).

*H10*: ASE. SL, AS, and MBW will statistically mediate the association between SI and students’ AP.

## 3 Materials and methods

### 3.1 Research design

The objective of this study is to ascertain and evaluate the determinants of individuals’ AP. Thus, it will validate the relationship through a cross-sectional study using a quantitative approach, as discussed in the literature (Shahzad et al., 2024; Yoon and Leem, 2021; Almulla and Al-Rahmi, 2023; Almulla, 2023).

### 3.2 Instruments and procedures

To measure the influence on the dependent variable AP, the current study employed a two-part online survey as the instrument. The first part was used to obtain demographic data, and the second part contained six variables, each with three or four items, from studies, all of which demonstrated proven validity and reliability (Table 1). The instrument used a five-point Likert scale ranging from one (strongly disagree) to five (strongly agree).

**Table 1:** Data collection instruments.

Variables included in the study	Items	Sources of the instruments
Social influence (SI)	3	Venkatesh et al. (2012), Strzelecki (2024), Korchak et al. (2025)
AI self-efficacy (ASE)	4	Carolus et al. (2023)
Smart learning (SL)	4	Shahzad et al. (2024), Zang et al. (2022)
Academic stress (AS)	4	Acosta-Enriquez et al. (2025), Zhang J. et al. (2024), Zhang S. et al. (2024), Zhang Y. et al. (2024)
Mental well-being (MWB)	4	Shahzad et al. (2024), Alam et al. (2021)
Academic performance (AP)	4	Shahzad et al. (2024), Whelan et al. (2020)

The internal consistency reliability of the measurement scales was assessed using Cronbach’s alpha, with one representative item presented for each construct to illustrate the content measured. Social Influence (SI), adapted from Venkatesh et al. (2012), Strzelecki (2024), and Korchak et al. (2025), was measured using three items, including “I have seen others using AI tools in both Arabic and English,” and demonstrated excellent reliability (*α* = 0.929). AI Self-Efficacy (ASE), sourced from Carolus et al. (2023), comprised four items, including “I can handle most problems related to artificial intelligence on my own,” and exhibited strong internal consistency (*α* = 0.851). Smart Learning (SL), adapted from Shahzad et al. (2024) and Zang et al. (2022), was measured using four items, one of which was “With smart technologies, I find myself engaging in group learning,” and showed acceptable reliability (*α* = 0.788). Academic Stress (AS), based on Acosta-Enriquez et al. (2025) and Zhang J. et al. (2024), Zhang S. et al. (2024), Zhang Y. et al. (2024), consisted of four items, including “I feel overwhelmed by the amount of academic work I have,” and demonstrated good internal consistency (*α* = 0.839). Mental Well-being (MWB), adapted from Shahzad et al. (2024) and Alam et al. (2021), was assessed using four items, including “I have been feeling optimistic,” and exhibited satisfactory reliability (*α* = 0.770). Finally, academic performance (AP), sourced from Shahzad et al. (2024) and Whelan et al. (2020), comprised four items, including “I am making progress in the quality of my studies,” and showed acceptable internal consistency (*α* = 0.808). Overall, all Cronbach’s alpha values exceeded the recommended threshold of 0.70 (Gefen et al., 2000), ranging from 0.770 to 0.929, thereby confirming that all scales possess satisfactory internal consistency reliability for the purposes of this study (Table 1 and Appendix A).

### 3.3 Participants

The sample included students from various higher education institutions in Saudi Arabia, including King Saud University, King Abdulaziz University, and the University of Jeddah. Data collection was conducted using convenience sampling, as it is quick, easy, and cost-effective (Acharya et al., 2013). An online survey was created using Google Forms and sent via email in February 2026. The email described the purpose of the research, briefly outlined the concepts involved, and highlighted the importance of participants’ views. The participants would be ensured a degree of anonymity, with no personal identifiers collected. The purpose was explained, along with the expected survey duration of approximately 10–15 min. This was a short, mobile-friendly survey to avoid non-response bias.

There were 354 respondents who participated. After screening, 9 multivariate outliers were identified and removed, resulting in 345 valid cases. Based on Hair et al. (2020), the appropriate sample size measure should be calculated through power analysis based on the regression models used. The current paper has used an *a priori* sample size calculator for the structural equation modelling (SEM) software package (Soper, 2020). This software requires the input of parameters like effect size, the degree of statistical power, the total number of observed (all the measurement items) and latent variables (both endogenous and exogenous constructs) present in the model, and the probability for identifying the minimal sample size of the SEM approach (Cohen, 2013; Westland, 2010). Therefore, with 29 observed variables and 6 latent variables, with an anticipated effect size (*d* = 0.30), the desired probability (*p* = 0.05), and the statistical power (0.95), the minimal sample size is 236 (Ferdowsi and Moradi, 2026). The sample of 345 respondents successfully met the required criteria.

### 3.4 Sample characteristics

Of the 345 respondents, 68.4% were male and 31.6% female. The largest age group was 19–21 years (67.5%). The majority were full-time students (88.1%), and most were seniors (43.8%). Domestic students made up 92.5% of the sample. Regarding monthly family income, 66.4% reported 15,000 SR or more. Table 2 outlines the profile of the study participants.

**Table 2:** Profile of respondents.

Variable	Category	Frequency (*n*)	Percentage (%)
Gender	Male	236	68.4
	Female	109	31.6
Age (years)	19–21	233	67.5
	22–24	111	32.2
	25–27	1	0.3
Employment status	Full-time student	304	88.1
	Part-time job	22	6.4
	In-campus job	8	2.3
	Full-time job	11	3.2
Enrollment status	Freshman	30	8.7
	Junior	84	24.3
	Sophomore	80	23.2
	Senior	151	43.8
Student type	International	26	7.5
	Domestic	319	92.5
Family income (SR)	Less than 2,000	10	2.9
	2,000–5,999	13	3.8
	6,000–9,999	34	9.9
	10,000–15,000	59	17.1
	15,000 or more	229	66.4

Author’s own creation.

### 3.5 Control variables

In an effort to strengthen the validity of the structural model and control for potential confounding factors, gender, age, employment, enrollment status, student type, and family income were identified as control variables that directly impact AP. The inclusion of these variables made it easier for the researcher to conduct hypothesis tests concerning the study variables. Among the control variables mentioned above, only student type had a significant impact on AP (*β* = −0.206, *t* = 1.702, *p* = 0.044). The negative coefficient indicates that students coded with the higher value of the student type variable exhibited lower AP than the reference group. On the other hand, none of the other control variables had a significant correlation with AP.

### 3.6 Statistical tools

The investigation used Structural Equation Modelling (SEM) through Partial Least Square (PLS) using SmartPLS 4.0. (Hair et al., 2020; Sharma et al., 2025). This technique was adopted since, unlike CB-SEM, it is not sensitive to the normality of the dataset, ensuring optimal relationships between indicators and latent variables, as well as between latent variables (Ringle et al., 2020), allows conducting exploratory research (Hair et al., 2020), works with small samples and non-normal data (Badghish et al., 2023), and suits the purpose of complex modelling (Kanth and Sinha, 2026). Hair et al. (2020) argue that there is a need for undertaking a two-staged analysis of the data. The process starts with assessing the measurement model for the constructs under investigation for their validity and reliability, followed by testing the structural model to confirm the study’s hypothesis (Oquaye et al., 2022). Data analysis involves reporting results on both the measurement model and the structural model using PLS (Rajpal and Singh, 2024).

## 4 Data analysis

### 4.1 Model robustness

#### 4.1.1 Common method bias

Common Method Variance refers to systematic error associated with the measure used which does not capture the actual tendencies of the respondents, hence resulting in an introduction of spurious variability in the respondents’ answers (Podsakoff et al., 2012). Common method variance is commonly reported where the researcher uses only one method for measuring more than one construct in a research study, possibly affecting the relationship between the constructs while also influencing the reliability and validity of the measures (Kock et al., 2021). The Harman one-factor test was conducted to assess common method bias, suggesting that a dominant factor accounted for 37.259% of the total variance. The observed value is significantly lower than the 50% threshold proposed by Podsakoff and Organ (1986), suggesting that common method bias is not a concern in this study. To further examine multicollinearity among the constructs, the Variance Inflation Factor (VIF) test was employed, following the approach recommended by Al Shbail et al. (2022). The results indicate moderate multicollinearity among constructs, as all VIF values remained below the critical limit of 10 (Thompson et al., 2017), ranging from 1.151 to 5.952 across the constructs (see Table 3).

**Table 3:** Model fit indices.

Measures of Indices	Preferred Range	Model Value	Remarks
SRMR	Less than 0.08	0.066	Accepted
NFI	Between 0 and 1, but > 0.9 considered good fit	0.776	Moderately accepted

#### 4.1.2 Model fit

The general applicability of the proposed theoretical model was assessed against the evaluation criteria. Fit indices such as SRMR and NFI were used to evaluate the goodness of fit of the developed model (Table 4). The obtained SRMR of 0.066 indicates that the model exhibits a good fit since this indicator lies below the cutoff point of 0.08. Also, the calculated NFI value of 0.776 shows an adequate fit for the research model (Table 3). In PLS-SEM analysis, the most widely used indicator of goodness-of-fit is SRMR, and this measure confirms a good model fit in this study (Hair et al., 2020). Next, this section highlights the *R*^2^ values for the endogenous constructs (see Table 4). *R*^2^ values of 0.75, 0.5, and 0.25 reflect strong, medium, and weak relationships, respectively. This study reports *R*^2^ values for five constructs—ASE, SL, AS, MBW, and AP at 0.455, 0.248, 0.210, 0.209, and 0.625, respectively. The results suggest that the proposed model exhibits moderate explanatory power, in line with the guidelines (Kanth and Sinha, 2026).

**Table 4:** Model explanatory power.

Constructs	*R*-square	*R*-square adjusted
AP	0.625	0.613
AS	0.210	0.206
ASE	0.455	0.454
MWB	0.209	0.204
SL	0.248	0.244

#### 4.1.3 PLS-predict

Predictive relevance and predictive power were assessed using the PLS-predict technique within the presented structural model. Following the guidelines set out by Hair et al. (2020). *Q*^2^ scores greater than 0.50, 0.25, and 0 correspond to large, medium, and low predictive relevance, respectively. As can be seen from the results, all the *Q*^2^ predict values for AP, AS, ASE, MWB, and SL constructs were higher than 0, indicating their predictive relevance (Guenther et al., 2023). Specifically, the average *Q*^2^ predict scores for AP, AS, ASE, MWB, and SL were 0.329, 0.098, 0.450, 0.163, and 0.188, respectively. Collectively, these findings suggest that the model possesses moderate predictive relevance and satisfactory predictive performance. Additionally, the PLS-SEM approach yielded lower RMSE and MAE than the LM and IA (Table 5 and Appendix B).

**Table 5:** PLS-predict.

Construct	*Q*^2^ predict (Avg)	RMSE (PLS)	RMSE (LM)	RMSE (IA)	Predictive power
ASE	0.321	0.771	0.774	0.929	Moderate
SL	0.114	1.014	1.026	1.078	Low
AS	0.067	1.012	1.027	1.047	Low
MWB	0.094	0.997	1.011	1.046	Low
AP	0.210	0.863	0.850	0.970	Low–moderate
Overall	0.161	0.931	0.938	1.014	Moderate

*Q*^2^ predict > 0 indicates predictive relevance. Predictive power is assessed by comparing RMSE (PLS) with RMSE (LM) and RMSE (IA). Higher *Q*^2^ predict values indicate stronger out-of-sample predictive capability. IA, naive benchmark; LM, linear model.

#### 4.1.4 Measurement model

The Structural Equation Modelling (SEM) measurement model was tested to ensure that the latent variables had sufficient reliability and validity (Kanth and Sinha, 2026; Biswas, 2024). Convergent validity was established since all constructs exceeded the acceptable criteria of outer loading, average variance extracted (AVE > 0.50), and composite reliability (CR > 0.70) (Hair et al., 2020). Cronbach’s alphas were found in the range of 0.770 and 0.929, which exceeded the acceptable threshold of 0.70 (Gefen et al., 2000), while the values of rho_a varied from 0.791 to 0.931 (Table 6). Discriminant validity was tested using the Fornell-Larcker approach (Fornell and Larcker, 1981) and the HTMT ratio. The square roots of AVE were higher than inter-construct correlations (Table 7), and all HTMT values were lower than 0.85 (Hair et al., 2020) (Table 8), which means that discriminant validity exists between the six latent variables. The measurement model is shown in Figure 2.

**Table 6:** Reliability analysis.

Latent variables	Reliability analysis	Outer loadings	VIF	CR (Composite reliability)	Cronbach’s alpha (*α*)	AVE (Average Variance Extracted)
AP	AP_1	0.685	1.363	0.822	0.808	0.640
	AP_2	0.875	2.563			
	AP_3	0.874	2.551			
	AP_4	0.750	1.456			
ASE	ASE_1	0.504	1.151	0.907	0.851	0.717
	ASE_2	0.948	5.952			
	ASE_3	0.916	3.894			
	ASE_4	0.936	4.726			
AS	AS_1	0.758	1.723	0.865	0.839	0.681
	AS_2	0.917	3.215			
	AS_3	0.894	2.839			
	AS_4	0.713	1.534			
MBW	MWB_1	0.788	1.548	0.792	0.770	0.593
	MWB_2	0.835	1.795			
	MWB_3	0.808	1.697			
	MWB_4	0.634	1.318			
SI	SI_1	0.932	3.405	0.931	0.929	0.876
	SI_2	0.933	3.800			
	SI_3	0.942	4.039			
SL	SL_1	0.681	1.294	0.791	0.788	0.614
	SL_2	0.825	1.832			
	SL_3	0.825	2.012			
	SL_4	0.795	1.682			

**Table 7:** Fornell–Larcker criterion.

Constructs	AP	AS	ASE	MWB	SI	SL
AP	0.800					
AS	−0.466	0.825				
ASE	0.643	−0.231	0.847			
MWB	0.524	−0.319	0.365	0.770		
SI	0.612	−0.324	0.675	0.413	0.936	
SL	0.588	−0.435	0.465	0.401	0.447	0.784

**Table 8:** HTMT ratio.

HTMT ratio	AP	AS	ASE	MWB	SI	SL
AP						
AS	0.557					
ASE	0.777	0.270				
MWB	0.655	0.393	0.454			
SI	0.698	0.365	0.753	0.472		
SL	0.733	0.533	0.570	0.521	0.524	

**Figure 2:**
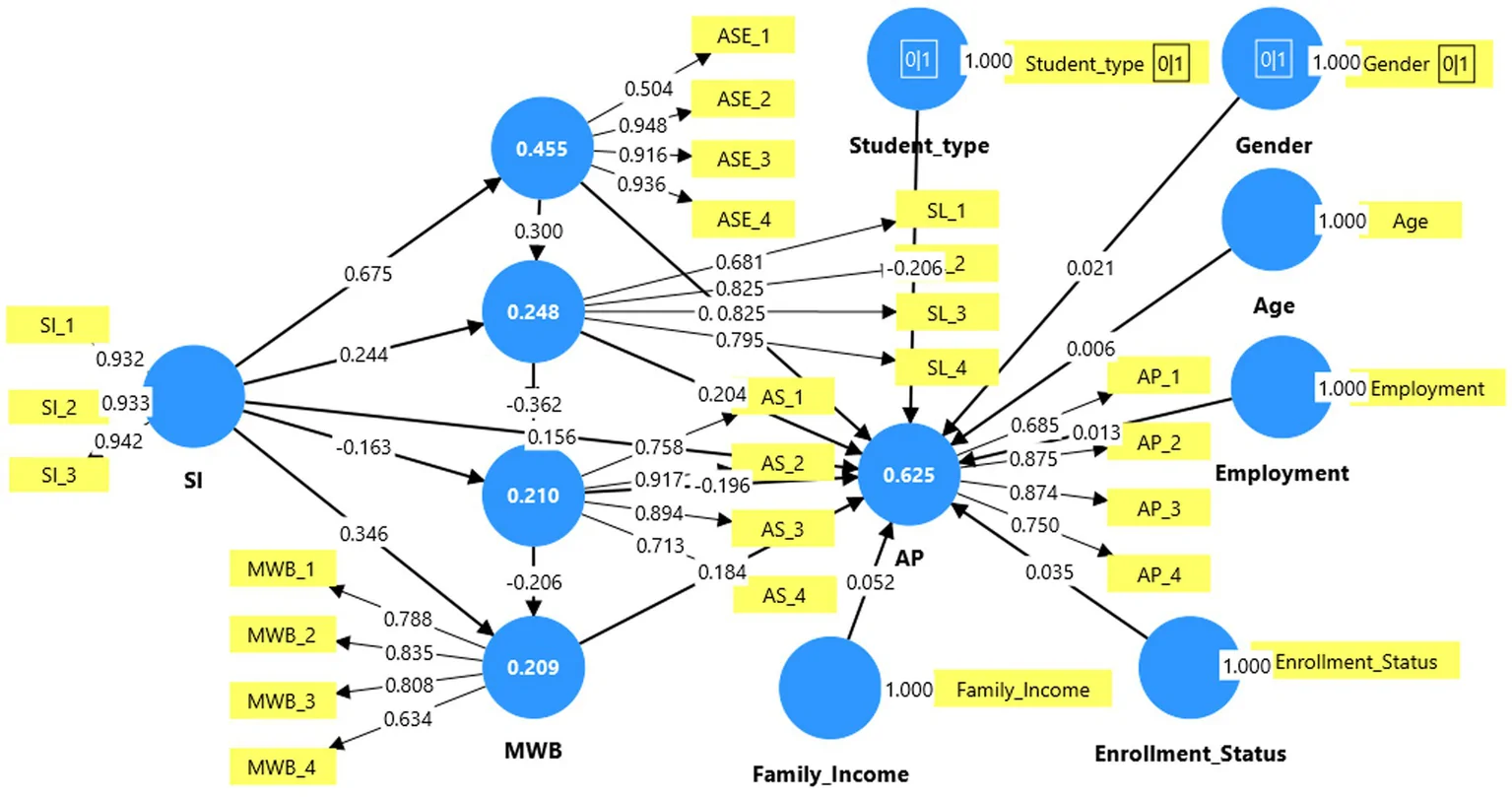
Measurement model.

#### 4.1.5 Structural model

Once reliability and validity are well established, bootstrapping with 5,000 resamples is used to calculate the β and t-values. The structural model is used to analyze the interrelationships among latent constructs through the patterns of relationships among observable indicators (Biswas, 2024). The initial hypothesis indicated that there would be a statistically significant correlation between SI and AP. According to the statistical outcomes, there is indeed a significant correlation (*β* = 0.156, *t* = 2.083, *p* < 0.05), supporting H1. Moreover, ASE positively affects AP (*β* = 0.331, *t* = 5.120, *p* < 0.001), which confirms H2. Further, the findings reveal that SL positively impacts AP (*β* = 0.204, *t* = 3.978, *p* < 0.001). Therefore, H3 is confirmed. Subsequently, AS demonstrates a substantial negative impact on AP (*β* = −0.196, *t* = 4.298, *p* < 0.001), thus confirming H4. Finally, MBW exhibits a substantial positive impact on AP (*β* = 0.184, *t* = 4.113, *p* < 0.001), thus corroborating H5. Furthermore, ASE is proposed to mediate the relationship between SI and AP. The results suggest a strong relationship (*β* = 0.223, *t* = 4.869, *p* < 0.001), thereby supporting H6. Also, H7 proposes that SL mediates the relationship between SI and AP (*β* = 0.050, *t* = 2.652, *p* < 0.05), thereby validating H7. In addition, the connection between SI and AP has been mediated by AS (*β* = 0.032, *t* = 2.191, *p* < 0.05), thereby reinforcing H8. In the same manner, the SI–AP connection has been mediated by MBW (*β* = 0.064, *t* = 3.019, *p* < 0.05), and thus, H9 has been confirmed. Moreover, ASE, SL, AS, and MBW have mediated the SI–AP relationship serially (*β* = 0.003, *t* = 1.674, *p* < 0.05), thus validating H10. All mediation and serial mediation effects were partial, as SI maintained a significant positive effect on AP (*β* = 0.156, *t* = 2.083, *p* < 0.05). The results of the hypothesis and mediation analyses are reported in Tables 9, 10, respectively, and the path diagram is shown in Figure 3.

**Table 9:** Hypothesis testing.

Hypothesis	Relationship	*β*	*t*-value	*p*-value	Result
H1	SI → AP	0.156	2.083	<0.05	Supported
H2	ASE → AP	0.331	5.120	<0.001	Supported
H3	SL → AP	0.204	3.978	<0.001	Supported
H4	AS → AP (−)	−0.196	4.298	<0.001	Supported
H5	MBW → AP	0.184	4.113	<0.001	Supported

**Table 10:** Mediation and serial mediation results.

Hypothesis	Indirect relationship	*β*	*t*-value	*p*-value	Result
H6	SI → ASE → AP	0.223	4.869	<0.001	Supported
H7	SI → SL → AP	0.050	2.652	<0.05	Supported
H8	SI → AS → AP	0.032	2.191	<0.05	Supported
H9	SI → MBW → AP	0.064	3.019	<0.05	Supported
H10	SI → ASE → SL → AS → MBW → AP	0.003	1.674	<0.05	Supported

**Figure 3:**
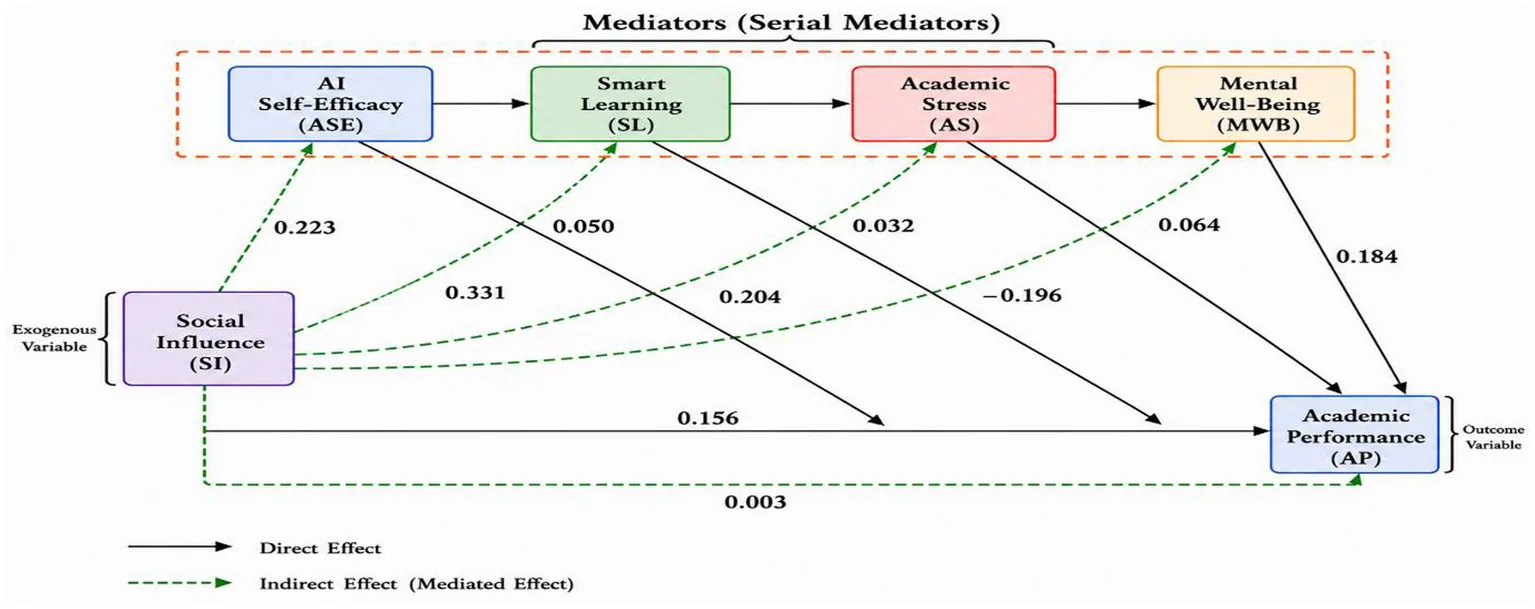
Path diagram.

## 5 Discussion

In this study, using SLT, a complete conceptual framework that captures the impact of SI on AP via individual and serial mediations through ASE, SL, AS, and MWB is formulated and validated. The next section examines the results from a higher education perspective and, in comparison, with past literature.

Initially, regarding hypothesis H1, the findings indicate a positive correlation between SI and AP (*β* = 0.156, *t* = 2.083, *p* < 0.05). This finding is consistent with previous studies showing a positive correlation between the two variables (Gallardo et al., 2016; Zhang and Qian, 2024; Song et al., 2015; Ganotice and King, 2014). A possible reason why SI could be beneficial to AP is that SI provides an environment in which students gain emotional and social support, making them more confident, resilient, and motivated learners (Zhang and Qian, 2024; Dupont et al., 2015; Dodeen and Hassan, 2021). However, it contradicts earlier findings showing either a weak or no correlation between the two variables (Dodeen and Hassan, 2021; Kirk, 2000).

Next, regarding hypothesis H2, the findings reveal a positive relationship between ASE and AP (*β* = 0.331, *t* = 5.120, *p* < 0.001), consistent with previous studies (Lotey et al., 2026; Chen Chen et al., 2025; Fung et al., 2024). Our analysis shows that ASE is the best predictor of AP, which is consistent with a recent study (Dang and Nguyen, 2026). This may be because students who score higher on the ASE use AI tools more effectively, overcome learning barriers, and achieve better learning outcomes. AI self-efficacy helps learners adopt effective learning strategies and solve problems encountered when using AI tools. Similarly, confident AI users achieve better learning outcomes, leading to better AP (Lotey et al., 2026; Chen Chen et al., 2025; Fung et al., 2024). However, this finding contradicts a recent study that reported a positive but non-significant relationship between the two variables (Be’cirovi’c et al., 2025).

Moreover, regarding hypothesis H3, the results reveal a positive relationship between SL and AP, consistent with earlier studies (Shahzad et al., 2024; Kaur et al., 2022; Chen and Liu, 2024; Yousaf et al., 2023). SL positively influences AP through the following aspects: personalization of the learning process, real-time feedback, opportunities for collaborative learning, and the availability of educational materials (Shahzad et al., 2024; Hilal et al., 2022). However, this finding is inconsistent with earlier research that identified a negative association between the two variables (Mantooth et al., 2021).

However, following, with regard to H4, the results show that there is a significant negative influence of AS on AP (*β* = −0.196, *t* = 4.298, *p* < 0.001), similar to previous studies (Lai and Ng, 2026; Ragusa et al., 2023; Amirkhan et al., 2020). This may happen since AS causes psychological strain, anxiety, and emotional exhaustion, making it difficult for students to concentrate and learn. High academic stress affects students’ cognitive processes and increases negative emotions, leading to poorer academic outcomes. Additionally, academic stress leads to academic exhaustion, making it difficult for students to perform academically (Ragusa et al., 2023). In contrast, our findings differ from previous studies that reported a dual (positive and negative) and no correlation between the two constructs (You, 2018; Pérez-Jorge et al., 2025; Broks et al., 2024; Ross et al., 2024). According to these, short-term stress may motivate students and enhance their performance under pressure; prolonged stress can adversely affect both health and AP (Pérez-Jorge et al., 2025; Ross et al., 2024).

Additionally, regarding H5, the findings revealed a significant positive effect of MBW on AP (*β* = 0.184, *t* = 4.113, *p* < 0.001), consistent with previous studies (Li et al., 2022; Chu et al., 2022; Monzonís-Carda et al., 2025). MBW will improve focus, motivation, resilience in the face of difficulties, and cognitive abilities that help with coping with academic tasks. High levels of stress and depression lead to a decrease in the ability to focus, learn, and perform academically, which results in poor grades and academic achievements (Shahzad et al., 2024; Chu et al., 2022). However, the present results deviate from previous research, which found both positive and negative relationships between these variables (Rüppel et al., 2015; Zhang J. et al., 2024). In one study, for instance, freshmen had lower MWB than fifth-year students but performed better academically. This observation indicates that as students become accustomed to the university setting, their mental wellbeing improves gradually (Zhang J. et al., 2024).

Moreover, with respect to H6, ASE plays a significant mediating role in the relationship between SI and AP (*β* = 0.223, *t* = 4.869, *p* < 0.001), a key novelty of this research. This is possible because SI enables students to implement AI technology effectively (Soodan et al., 2024; Li et al., 2024), and ASE positively affects students’ academic performance (Shahzad et al., 2025; Singh et al., 2025). Thus, SI indirectly improves AP by strengthening ASE.

In addition, regarding H7, the findings indicate that SL mediates the association between SI and AP (*β* = 0.050, *t* = 2.652, *p* < 0.05), consistent with prior literature (Shahzad et al., 2024). From a social constructivist perspective, SI facilitates learning through cooperation and interaction between students and instructors, leading to better learning outcomes (Gao et al., 2024; Wang et al., 2022; Pan et al., 2024). Moreover, the use of SL also facilitates student cooperation and improves AP (Boer et al., 2021).

Also, regarding H8, the results show that AS plays an important role as a mediator in the association between SI and AP (*β* = 0.032, *t* = 2.191, *p* < 0.05). This is another innovative aspect of the present study. SI, with the help of family, peers, and teachers, helps to create a favorable educational climate and decrease AS (Puteri and Dewi, 2021). As higher AS has a negative impact on resilience, the learning process, and AP (You, 2018; Pascoe et al., 2020; Al-Roomy, 2025; Iqra, 2024; Lai and Ng, 2026; Deng et al., 2022), then high SI will increase AP indirectly by reducing AS.

The same applies to the association of H9_,_ since the findings reveal that MWB significantly mediates the association between SI and AP (*β* = 0.064, *t* = 3.019, *p* < 0.05), consistent with existing literature (Gai et al., 2026; Chen et al., 2023). Positive SI increases students’ MWB by fostering healthy social connections and meeting psychological needs (Umberson and Montez, 2010). This means that SI indirectly increases AP by increasing MWB, as improved MWB enhances engagement and motivation, leading to higher AP (Agha et al., 2023; Reuter and Forster, 2021; Kilgore et al., 2023).

Finally, with regard to H10, the findings reveal that ASE, SL, AS, and MWB serve as mediators between SI and AP in sequence (*β* = 0.003, *t* = 1.674, *p* < 0.05), thus making yet another unique contribution to this study. Although the serial mediation effect is small (*β* = 0.003), its statistical significance indicates that the proposed sequential pathway provides meaningful theoretical evidence supporting the relationships among the study constructs. SI is linked with fostering collaborative and SL (Shahzad et al., 2024), which, in turn, positively impacts mental wellbeing (Hu et al., 2026) and ASE (Marques-Pinto et al., 2025), reduces AS (Wang and Xu, 2026) and, finally, boosts AP (Pascoe et al., 2020; Al-Roomy, 2025; Iqra, 2024; Lai and Ng, 2026; Deng et al., 2022).

## 6 Theoretical contribution and practical implications

This study holds significant theoretical implications. First, it highlights the complex relationships among SI, ASE, SL, AS, MWB, and AP, thereby enhancing understanding of the mechanisms linking these constructs. Secondly, this paper empirically demonstrates that SI has an indirect influence on AP through the mediating and serial mediating roles of ASE, SL, AS, and MWB, thus highlighting the importance of social variables in determining students’ AP. Following the principles of Social Learning Theory, individuals learn from social interactions and observations (Bandura and Walters, 1977). Therefore, SI improves students’ motivation towards AI-based learning practices (Almulla and Al-Rahmi, 2023) and ASE (Bewersdorff et al., 2025; Shahzad et al., 2025), encourages smart learning (Shahzad et al., 2024), reduces AS, and, at the same time, increases their MBW (Zhang J. et al., 2024; Zhang S. et al., 2024; Zhang Y. et al., 2024; Barbayannis et al., 2022), thereby increasing AP.

Apart from the theoretical contribution, this study has several practical implications for educators, institutions of higher education, and policymakers. Firstly, educational institutions are recommended to foster positive SI among students through collaborative learning, peer mentoring, and a supportive learning environment to stimulate AI implementation and improve students’ academic achievement (Acosta-Enriquez et al., 2025). Secondly, educators are advised to incorporate AI-based learning tools into the teaching process and to motivate students to use AI for problem-solving, autonomous learning, and formative feedback, rather than just for completing assignments. They are also advised to implement AI literacy training and AI-based instructions to promote ASE and responsible usage of AI (Shahzad et al., 2024; Zhang J. et al., 2024; Zhang S. et al., 2024; Zhang Y. et al., 2024; Zhu, 2026). Thirdly, policymakers are expected to create AI integration policies and guidelines aimed at promoting the ethical use of AI, personalized curricula, data privacy, equal opportunities, and appropriate funding for AI-based education. Fourthly, educators are suggested to utilize peer workshops, student ambassador programs, and collaborative learning to increase AI engagement, motivation, knowledge exchange, and SL behaviors (Almulla, 2023; Korchak et al., 2025). Finally, educational institutions should develop wellbeing programs such as stress management, resilience building, and psychological counseling while promoting responsible AI usage and improving AI reliability through cooperation of educators, policymakers, technologists, and psychologists to improve the MWB and AP of students.

## 7 Limitations and future research directions

Despite its valuable contribution to the field of research, there are certain drawbacks that future researchers might use as guidance. The first one concerns the use of convenience sampling, and the sample size is quite small. Convenience sampling involves selecting participants based on their accessibility and willingness to participate in the research, without using randomization. This approach increases the possibility of sampling bias and makes the sample less representative. Thus, it limits the generalizability of the results (Alzahrani and Bhunia, 2026). Second, the gender imbalance (68.4% male and 31.6% female) may limit the generalizability of the findings across gender groups. Future studies should employ more balanced or stratified sampling to ensure adequate representation of both genders. Future research could also examine gender as a moderating variable to determine whether AI perceptions, learning behavior, and academic performance differ by gender. The third drawback is that this research has been carried out at only three public universities in Saudi Arabia. To increase the external validity of the results, future research should include a wider range of national settings. The fourth limitation is common method variance, which can occur when only a single questionnaire is used. However, common method variance did not occur in the current research; still, it is possible in the future. Using more varied data-gathering methods in future research can help avoid this problem. Finally, no moderators have been considered for the relationships among the variables of interest. It is crucial to conduct further studies to identify new mediators and predictors of the processes in question. For instance, one can study potential mediators, such as academic engagement and AI literacy, to explain how social influence affects performance, while time management would help explain why smart learning reduces academic stress. Among the possible moderators, there might be gender and academic field, since it would be interesting to understand for whom, the discussed effects occur more strongly.

## 8 Conclusion

The proposed study develops and validates a unified model based on the principles of Social Learning Theory to identify the mechanisms by which SI increases AP among students via ASE, SL, AS, and MWB. All 10 hypotheses are validated, showing that social influence affects the academic performance of Saudi university students in both direct and indirect ways through different individual and sequential mediation processes, with ASE identified as the best predictor of AP. The theoretical contribution of the study is its extension of the Social Learning Theory into an educational setting where AI is also used, while its practical value is related to the development of guidelines for educators and policymakers concerning the creation of positive social environments, raising awareness on AI, implementing smart learning systems and programs aimed at improving MWB. The limitations of the study include its cross-sectional design, the convenience sampling approach used to collect data from three public universities, and its self-reported nature, which limits its generalizability and the conclusions drawn.

## Data Availability

The raw data supporting the conclusions of this article will be made available by the authors, without undue reservation.
