# Supplementary material for: Social influence and academic performance: a serial mediation model in AI-enhanced learning
Source: Front Psychol. 2026 Sep 18;17:1928799. doi: 10.3389/fpsyg.2026.1928799 (PMC13630773; doi:10.3389/fpsyg.2026.1928799)
Supplement: Supplementary file 1 [file Supplementary_file_1.docx]

Appendix A. Survey questionnaire

| Construct | Code | Measurement Item |
| --- | --- | --- |
| Social Influence (SI) | SI1 | I have seen others using AI tools in both Arabic and English. |
|  | SI2 | I will use AI tools after seeing my friends using them in both Arabic and English. |
|  | SI3 | Other students seemed interested in AI tools when they saw me using them in both Arabic and English. |
| AI Self-Efficacy (ASE) | ASE1 | I can rely on my skills in difficult situations when using AI. |
|  | ASE2 | I can handle most problems related to artificial intelligence on my own. |
|  | ASE3 | I stay up-to-date with the latest innovations in AI applications. |
|  | ASE4 | Despite the rapid changes in AI, I can always keep up to date. |
| Smart Learning (SL) | SL1 | With smart technologies, I find myself learning diverse topics. |
|  | SL2 | With smart technologies, I find myself engaging in group learning. |
|  | SL3 | With smart technologies, I find myself performing better than others. |
|  | SL4 | With smart technologies, I find myself enjoying learning about new topics. |
| Academic Stress (AS) | AS1 | I feel overwhelmed by the amount of academic work I have. |
|  | AS2 | I experience anxiety when facing academic deadlines. |
|  | AS3 | I worry about not being able to meet academic expectations. |
|  | AS4 | Academic stress affects my ability to concentrate. |
| Mental Well-Being (MWB) | MWB1 | I have been feeling optimistic. |
|  | MWB2 | I have been feeling relaxed. |
|  | MWB3 | I have been dealing with problems well. |
|  | MWB4 | I have been thinking clearly. |
| Academic Performance (AP) | AP1 | I am making progress in my quantity of study. |
|  | AP2 | I am making progress in the quality of my studies. |
|  | AP3 | I am making progress in my personal career goals. |
|  | AP4 | I am making progress in developing the skills needed for my future career. |

Appendix B. Indicator-level PLS-predict assessment

| Indicator | Q²predict | PLS-SEM RMSE | PLS-SEM MAE | LM RMSE | LM MAE | IA RMSE | IA MAE |
| --- | --- | --- | --- | --- | --- | --- | --- |
| ASE_1 | 0.116 | 1.069 | 0.878 | 1.072 | 0.876 | 1.137 | 0.950 |
| ASE_2 | 0.383 | 0.675 | 0.447 | 0.677 | 0.452 | 0.859 | 0.715 |
| ASE_3 | 0.376 | 0.690 | 0.476 | 0.697 | 0.480 | 0.874 | 0.735 |
| ASE_4 | 0.410 | 0.651 | 0.442 | 0.648 | 0.438 | 0.847 | 0.706 |
| SL_1 | 0.135 | 1.003 | 0.784 | 1.010 | 0.776 | 1.078 | 0.875 |
| SL_2 | 0.109 | 0.995 | 0.781 | 1.003 | 0.781 | 1.054 | 0.863 |
| SL_3 | 0.110 | 1.020 | 0.806 | 1.038 | 0.824 | 1.082 | 0.876 |
| SL_4 | 0.103 | 1.039 | 0.804 | 1.053 | 0.822 | 1.097 | 0.881 |
| AS_1 | 0.052 | 1.008 | 0.811 | 1.023 | 0.818 | 1.035 | 0.816 |
| AS_2 | 0.088 | 0.943 | 0.766 | 0.956 | 0.773 | 0.988 | 0.767 |
| AS_3 | 0.081 | 1.001 | 0.809 | 1.019 | 0.811 | 1.045 | 0.834 |
| AS_4 | 0.045 | 1.095 | 0.902 | 1.108 | 0.902 | 1.120 | 0.906 |
| MWB_1 | 0.119 | 1.012 | 0.776 | 1.022 | 0.785 | 1.078 | 0.861 |
| MWB_2 | 0.133 | 0.899 | 0.714 | 0.925 | 0.724 | 0.965 | 0.786 |
| MWB_3 | 0.110 | 0.948 | 0.736 | 0.953 | 0.741 | 1.005 | 0.800 |
| MWB_4 | 0.014 | 1.127 | 0.909 | 1.142 | 0.919 | 1.135 | 0.923 |
| AP_1 | 0.094 | 0.992 | 0.768 | 0.968 | 0.776 | 1.042 | 0.843 |
| AP_2 | 0.303 | 0.774 | 0.574 | 0.765 | 0.532 | 0.927 | 0.738 |
| AP_3 | 0.267 | 0.785 | 0.574 | 0.780 | 0.541 | 0.916 | 0.730 |
| AP_4 | 0.176 | 0.902 | 0.666 | 0.887 | 0.663 | 0.994 | 0.804 |
